# Single Amino Acid Substitution in Loop1 Switches the Selectivity of α-Conotoxin RegIIA towards the α7 Nicotinic Acetylcholine Receptor

**DOI:** 10.3390/md22090390

**Published:** 2024-08-29

**Authors:** Jinpeng Yu, Junjie Xie, Yuting Ma, Pengcheng Wei, Panpan Zhang, Zepei Tang, Xiaopeng Zhu, Dongting Zhangsun, Sulan Luo

**Affiliations:** 1 Guangxi Key Laboratory of Special Biomedicine, School of Medicine, Guangxi University, Nanning 530004, China; 2128391011@st.gxu.edu.cn (J.X.); yutingma2022@163.com (Y.M.); weipengcheng@gxu.edu.cn (P.W.); 2128391043@st.gxu.edu.cn (P.Z.); 13878604135@163.com (Z.T.); zhuxiaopeng@gxu.edu.cn (X.Z.); zhangsundt@163.com (D.Z.); 2 Key Laboratory of Tropical Biological Resources of Ministry of Education, Hainan University, Haikou 570228, China

**Keywords:** α-conotoxin RegIIA, selectivity, mutant, electrophysiology

## Abstract

α-Conotoxins are disulfide-rich peptides obtained from the venom of cone snails, which are considered potential molecular probes and drug leads for nAChR-related disorders. However, low specificity towards different nAChR subtypes restricts the further application of many α-conotoxins. In this work, a series of loop1 amino acid-substituted mutants of α-conotoxin RegIIA were synthesized, whose potency and selectivity were evaluated by an electrophysiological approach. The results showed that loop1 alanine scanning mutants [H5A]RegIIA and [P6A]RegIIA blocked rα7 nAChR with IC_50_s of 446 nM and 459 nM, respectively, while their inhibition against rα3β2 and rα3β4 subtypes was negligible, indicating the importance of the fifth and sixth amino acid residues for RegIIA’s potency and selectivity. Then, second-generation mutants were designed and synthesized, among which the analogues [H5V]RegIIA and [H5S]RegIIA showed significantly improved selectivity and comparable potency towards rα7 nAChR compared with the native RegIIA. Overall, these findings provide deep insights into the structure–activity relationship of RegIIA, as well as revealing a unique perspective for the further modification and optimization of α-conotoxins and other active peptides.

## 1. Introduction

Nicotinic acetylcholine receptors (nAChRs) are important members of the Cys-loop superfamily, which are pentameric ligand-gated ion channels and are formed with 17 distinct subunits (α1–α10, β1–β4, γ, δ, and ε) [1]. They can be divided into muscle-type (α1β1δγ and α1β1δε) and neuronal nAChRs (all other subtypes). Some α subunits can form homo-pentameric receptors, such as α7 nAChR, while other neuronal nAChR subtypes act as functional hetero-pentamers consisting of α and β subunits, such as α3β2, α3β4, etc. [1,2].

These various combinations of subunits produce different nAChR subtypes with diverse pharmacological and biophysical properties in vivo [3]. The results of previous studies showed that α3β2 nAChR regulates the morphology of human bronchial epithelial cells [4]. It also inhibits the release of glutamate from primary afferent C-fibers, resulting in reduced sensitivity to nociceptive mechanical stimulation [5]. α3β4 nAChR has been found to play a vital role in lung cancer, drug addiction, and nicotine-induced seizures [6]. Furthermore, several studies have shown that α3β4 nAChR is implicated in nicotinic-induced decreases in food intake and might be a potential target for treating food overconsumption and obesity [7]. α7 nAChR exhibits the characteristics of rapid activation and desensitization because of its high Ca^2+^ permeability [8]. This subtype is widely found in the central nervous system and is considered to be a potential target for the treatment of cognitive deficits, including Alzheimer’s disease, schizophrenia, and depression [9]. α7 nAChR is also expressed in non-neuronal cells such as immune cells, in which it participates in the cholinergic anti-inflammatory pathway [10]. It has also been proven to contribute to the aggressiveness of lung cancer cells [11], as well as playing an important role in regulating the signaling pathway of nicotine or choline-activated glioblastoma cell proliferation [12]. Therefore, specific ligands (agonists or antagonists) could be essential tools for the assessment of the physical functions and pharmacological activities of particular nAChR subtypes.

Conotoxins are bioactive peptides isolated from the venom of cone snails. Conotoxins have been classified into several pharmacological families according to their targets (α, γ, δ, ε, ι, κ, μ, ρ, σ, τ, χ, and ω) [13]. Among the different pharmacological classes, α-conotoxins are a family consisting of small peptides that act as antagonists of nAChRs. They share the typical CC-Xm-C-Xn-C framework, with “C” indicating Cys. According to the number of amino acids in the Cys-loops (loop1 and loop2), α-conotoxins can be further classified into many subfamilies (α4/7, α4/6, α4/5, α4/4, and α4/3) [14]. In consideration of their high potency and selectivity towards distinct nAChR subtypes, α-conotoxins could be valuable probes for the functional evaluation of nAChRs.

RegIIA is a typical α4/7-conotoxin identified from *Conus regius* that exhibits high activity towards α3β2, α3β4, and α7 nAChRs [15]. However, low specificity towards specific nAChR subtypes restricts the further application of this conotoxin. In previous research, Asn11 and Asn12 were identified as critical points for the α3β4 nAChR preference of RegIIA based on an alanine-scanning approach of loop2 amino acid residues, while the aromatic Asn9 substitution significantly enhanced its selectivity toward α3β2 nAChR [16,17]. The effects of RegIIA’s loop1 remain unknown.

Most studies on α-conotoxins only focus on the amino acid residues contained in loop2, because the primary structure is highly variable in loop2 and is presumed to be critical for α-conotoxins’ selectivity, while it is conserved in loop1, and is considered to play an important role when binding with receptors [18]. However, some evidence has also suggested that the amino acid composition in loop1 is not only related to α-conotoxins’ activity, but also has significant effects on their selectivity [19,20]. Here, we investigated the effects of loop1 on RegIIA’s activity and selectivity using alanine scanning and single amino acid substitution methods, after which, the potencies of analogs against rα3β4, rα3β2, and rα7 nAChRs were evaluated by electrophysiological approaches and compared with the native one. The stability of optimized analogs was evaluated in human serum. Molecular docking also provided a possible explanation for the selectivity shift led by mutations.

## 2. Results

### 2.1. Synthesis and Identification of the Native RegIIA and Its Analogues

**Figure 1:**
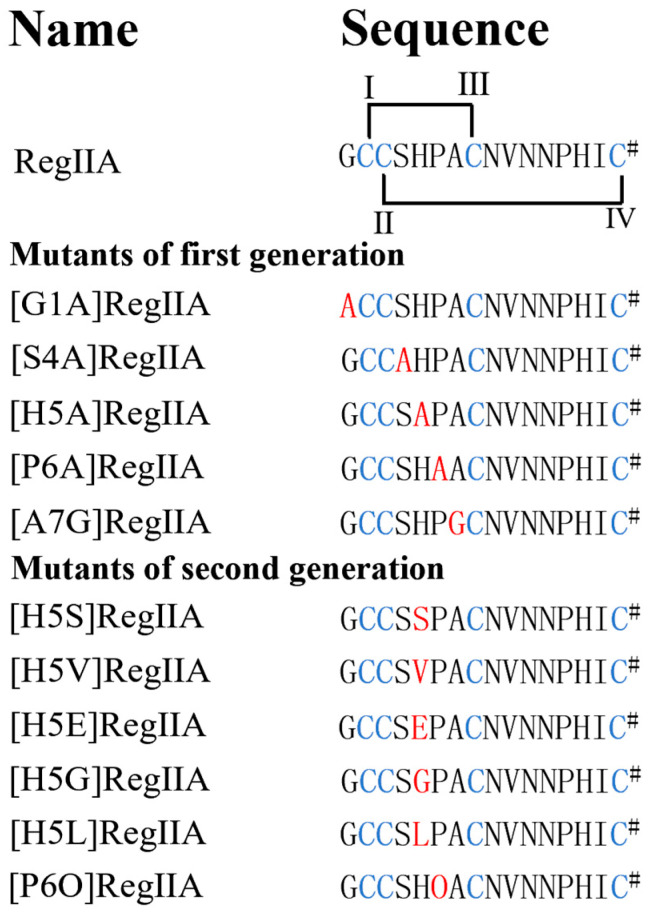
Sequences of the native RegIIA and its analogues. Conserved cysteine residues are marked in blue. Disulfide connectivity (I–III, II–IV) is indicated by lines connecting the Cys residues. The amino acids substituted in mutants are marked in red. # indicates the C-terminal amide. O, hydroxyproline (Hyp).

Because the native RegIIA exhibits a typical “globular” structure with the disulfide connectivity of Cys I–III and Cys II–IV, its analogues were all synthesized with this conformation by a two-step oxidative folding strategy (Figure 1). The molecular mass and purities (>95%) of mature peptides were identified by analytical reversed-phase high-performance liquid chromatography (RP-HPLC) and electrospray ionization mass spectroscopy (ESI-MS) (Figures S1 and S2).

### 2.2. Alanine Scanning of Loop1 Revealed His5 and Pro6 as a Critical Point for RegIIA’s Selectivity

**Figure 2:**
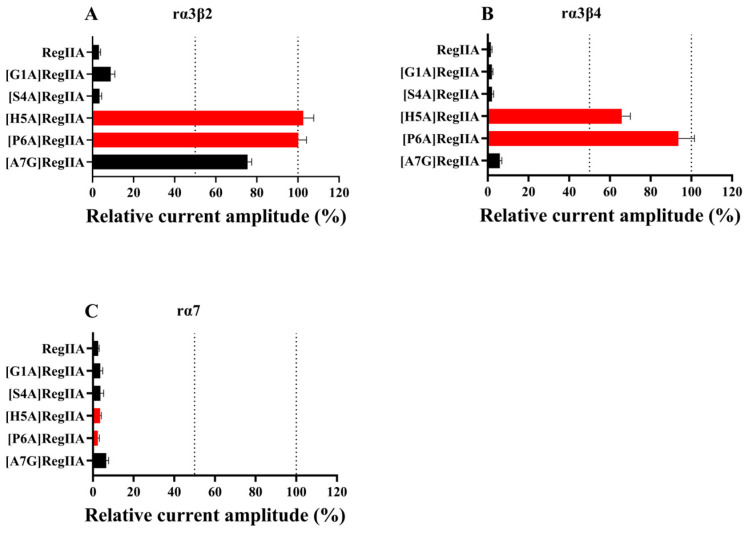
Relative current amplitude (%) of RegIIA and its first-generation mutants at a concentration of 10 μM on rα3β2 (**A**), rα3β4 (**B**), and rα7 (**C**). The inhibition of 10 μM [H5A]RegIIA and [P6A]RegIIA (both marked in red) were less than 50% on rα3β2 and rα3β4 subtypes, which were comparable with that of the native RegIIA on rα7 nAChR. All data were conducted at a holding potential of −70 mV and analyzed from 6–8 separate oocytes.

**Figure 3:**
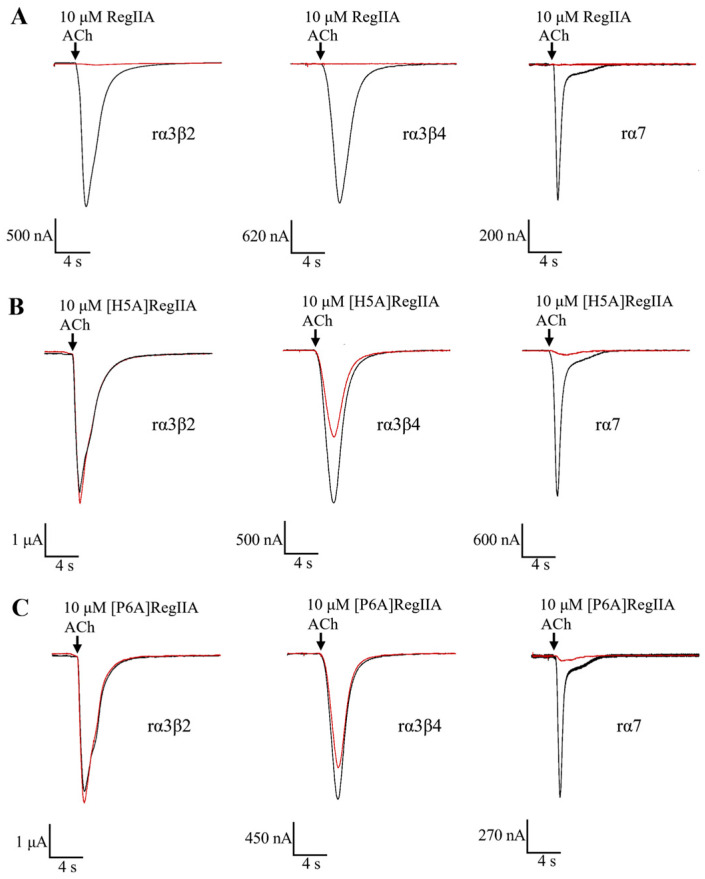
α-Conotoxin RegIIA (**A**), as well as its analogs [H5A]RegIIA (**B**) and [P6A]RegIIA (**C**), differentially blocking the current mediated by the rα3β2, rα3β4, and rα7 nAChR subtypes. Superimposed representative ACh-evoked currents were recorded from *Xenopus laevis* oocytes expressing rα3β2, rα3β4, and rα7 nAChRs in the absence (black trace) and presence (red trace) of incubation with 10 μM RegIIA or its analogs.

**Figure 4:**
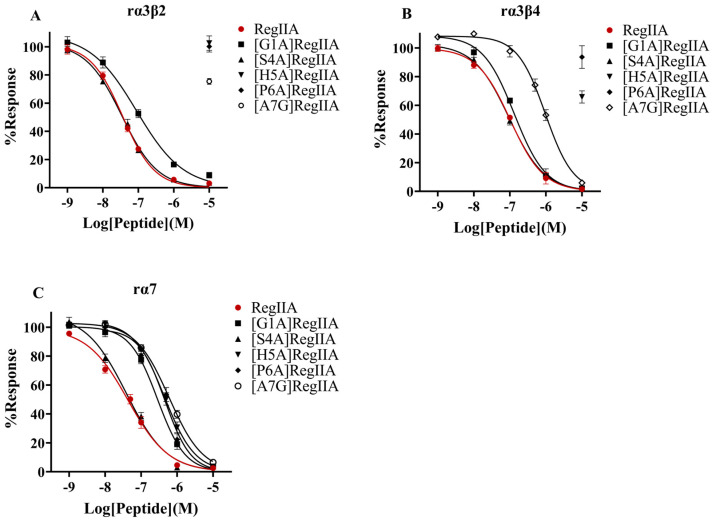
Concentration–response curves for the inhibition of the rα3β2 (**A**), rα3β4 (**B**), and rα7 nAChR (**C**) by RegIIA and its first-generation mutants. The concentration–response curves of [H5A]RegIIA and [P6A]RegIIA towards rα7 nAChR are shifted to the right compared to the native RegIIA, while their inhibition on the rα3β2 and rα3β4 subtypes were not obvious. All data points represent mean ± SEM values from 6–8 separate oocytes.

In order to determine the effects of the amino acid residues in loop1 on RegIIA’s potency and selectivity, we designed and synthesized a series of alanine-scanning mutants from RegIIA’s loop1, while the Ala7 residue was replaced by Gly (Figure 1). The potencies of all peptides were characterized on rα3β2, rα3β4, and rα7 nAChRs expressed on *Xenopus* oocytes (Figure 2, Figure 3 and Figure 4, Table 1). As the results showed, native RegIIA inhibited the rα3β2, rα3β4, and rα7 nAChR with IC_50_ values of 36 nM, 102 nM, and 43 nM, respectively. Most first-generation mutations led to different degrees of potency decreases, with the exception of the S4A substitution, which showed no obvious effect on RegIIA’s activity. [G1A]RegIIA showed comparable inhibitions on the rα3β2 and rα3β4 subtypes, while its IC_50_ towards rα7 nAChR was 286 nM, which was six-fold more compared with that of the native one. A7G substitution resulted in a complete loss in activity targeting rα3β2 nAChRs, as well as a substantial decrease in activity in both the rα3β4 and rα7 subtypes. Remarkably, the substitution of His5 or Pro6 with alanine significantly increased the selectivity for rα7 nAChR relative to that of the native RegIIA. The IC_50_ values of [H5A]RegIIA and [P6A]RegIIA on rα7 nAChR are 446 nM and 459 nM, respectively, which were ~10-fold less potent than RegIIA. However, their inhibition activity on rα3β2 and rα3β4 nAChRs was abolished at a concentration of 10 μM. As a result, both [H5A]RegIIA and [P6A]RegIIA showed >21-fold higher selectivity towards rα7 versus the other two subtypes, which was significantly improved compared with the native subtype.

### 2.3. H5S and H5V Mutation Led to Significant Selectivity Increase of RegIIA towards rα7 nAChR

**Figure 5:**
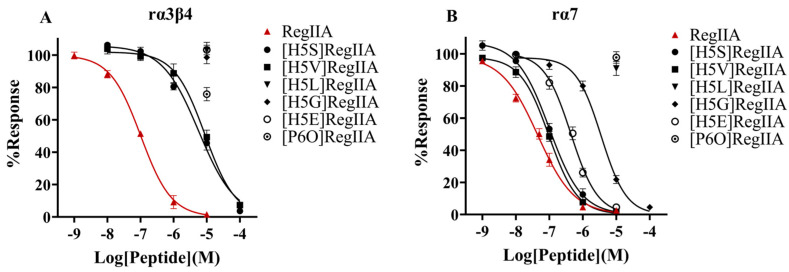
Concentration–response curves for the inhibition of the rα3β4 (**A**) and rα7 nAChR (**B**) by RegIIA’s second-generation mutants. The second-generation mutants showed substantial loss in potency towards all three nAChR subtypes, except for [H5S]RegIIA and [H5V]RegIIA, which exhibited only a ~2-fold potency decrease towards rα7 nAChR and >56-fold potency decrease towards rα3β2 and rα3β4 nAChRs, suggesting a >24-fold increase in selectivity compared with the native RegIIA. All data points represent mean ± SEM values from 6–8 oocytes.

As described above, His5 and Pro6 of loop1 were identified as determinants for the selectivity of RegIIA. We further designed and synthesized second-generation mutants by replacing His5 or Pro6 with other amino acid residues, and examined their activities against the rα3β2, rα3β4, and rα7 nAChR subtypes (Figure 1 and Figure 5, Table 2). As the results showed, all second-generation mutants showed a substantial potency decrease towards rα3β2 and rα3β4 nAChRs, while mutations P6O and H5L led to a complete loss in activity for all three nAChR subtypes. It is noteworthy that [H5S]RegIIA and [H5V]RegIIA showed the most significant selectivity increase among the second-generation mutants, which were ~two-fold less potent towards rα7 (IC_50_s were 100 and 97 nM, individually) and >56-fold less potent towards the other two subtypes relative to the native RegIIA, resulting in a >24-fold increase in selectivity.

### 2.4. Molecular Modeling of RegIIA Binding to Rat α3β2, α3β4, α7 nAChRs

**Figure 6:**
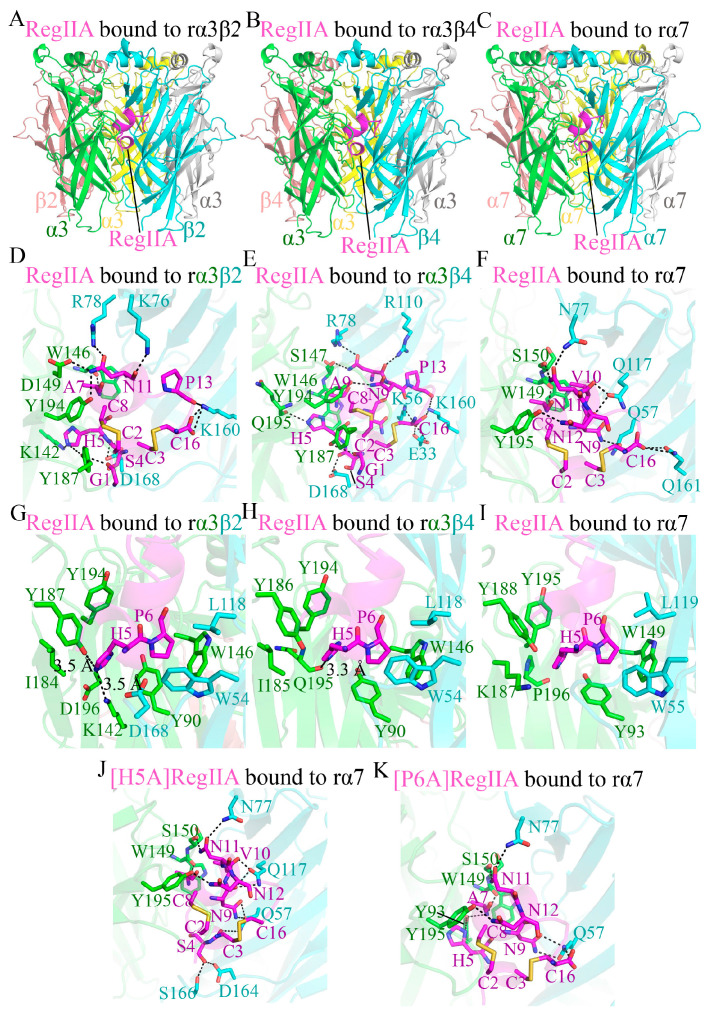
Interactions between RegIIA and its mutants with the extracellular domains (ECDs) of rα3β2, rα3β4, and rα7 nAChRs. (**A**) The overall structure of RegIIA binding to the ECD of the rα3β2 nAChR. (**B**) The overall structure of RegIIA binding to the ECD of the rα3β4 nAChR. (**C**) The overall structure of RegIIA binding to the ECD of the rα7 nAChR. (**D**) Detailed interactions of RegIIA with the rα3β2 nAChR, displaying all hydrogen bonds. Disulfide bonds between the side chains of C2–C8 and C3–C16 are shown. (**E**) Detailed interactions of RegIIA with the α3β4 nAChR, showing all hydrogen bonds. Disulfide bonds between the side chains of C2–C8 and C3–C16 are indicated. (**F**) Detailed interactions of RegIIA with the α7 nAChR, illustrating all hydrogen bonds. Disulfide bonds between the side chains of C2–C8 and C3–C16 are displayed. (**G**) Detailed interactions of RegIIA H5 and P6 with the α3β2 nAChR. Hydrogen bonds are formed between the side chain of H5 of RegIIA and the side chains of K142 and Y187 of the α3 subunit. (**H**) Detailed interactions of RegIIA H5 and P6 with the α3β4 nAChR. Hydrogen bonds are formed between the side chain of H5 of RegIIA and the oxygen of the main chain of Q195 of the α3 subunit. (**I**) Detailed interactions of H5 and P6 of RegIIA with the α7 nAChR. No hydrogen bonds are formed between H5 and the surrounding amino acids. (**J**) Interactions of the [H5A]RegIIA with the α7 nAChR, displaying all hydrogen bonds. Disulfide bonds between the side chains of C2–C8 and C3–C16 are shown. (**K**) Interactions of the [P6A]RegIIA with the α7 nAChR, illustrating all hydrogen bonds. Disulfide bonds between the side chains of C2–C8 and C3–C16 are indicated. Hydrogen bonds are represented by black dashed lines, with the cutoff set at 3.5 Å. Blue, Nitrogen; Red, Oxygen; Yellow, Sulfur.

We performed molecular modeling of RegIIA, [H5A]RegIIA, and [P6A]RegIIA bound to the extracellular domains (ECDs) of rα3β2, rα3β4, and rα7 nAChRs based on AlphaFold 3, respectively [21]. By comparison of our built model with previously reported models of RegIIA binding to nAChRs, we observed a similar binding mode (Figure 6A–C) [20,22,23,24], which indicates the validity of our constructed model. Structural analysis shows that RegIIA adopts a similar conformation when interacting with rα3β2, rα3β4, and rα7 nAChRs (Figure 6A–C).

In the interactions of RegIIA with rα3β2, rα3β4, and rα7, 13, 15, and 10 hydrogen bonds were formed, respectively (Figure 6D–F). Further structural analysis revealed that in the interaction between RegIIA and rα3β2 nAChR, the side chain of the H5 of RegIIA forms two hydrogen bonds with the side chain of the K142 and Y187 of the rα3 subunit (Figure 6G). In contrast, in the interaction with rα3β4, the side chain of the H5 of RegIIA forms a single hydrogen bond with the main chain oxygen of Q195 in the rα3 subunit (Figure 6H). However, the H5 of RegIIA does not participate in hydrogen bond formation with rα7 (Figure 6I). This partially explains why, in electrophysiological experiments, the [H5A]RegIIA, [H5V]RegIIA, and [H5S]RegIIA mutants still effectively inhibited rα7 nAChR activation, but almost lost the ability to inhibit rα3β2 and rα3β4 nAChR activation. Further molecular modeling confirmed that the [H5A]RegIIA mutant could bind to the rα7 nAChR similarly to RegIIA (Figure 6J).

Analysis of the P6 binding pocket of RegIIA shows that the binding pockets formed by rα3β2 and rα3β4 nAChRs are smaller (Figure 6G,H), whereas the pocket formed by rα7 nAChR is larger (Figure 6I), better accommodating changes caused by mutations at the P6 position of RegIIA. This also explains why, in electrophysiological experiments, the [P6A]RegIIA mutant lost the ability to inhibit rα3β2 and rα3β4 nAChR activation, but still effectively inhibited rα7. Molecular modeling of [P6A]RegIIA with rα7 nAChR showed that it binds to rα7 in a conformation similar to RegIIA (Figure 6K). Although the α7 nAChR has a larger binding pocket compared to rα3β2 and rα3β4 nAChRs, allowing for mutations at the P6 position of RegIIA, it remains insufficient to accommodate the [P6O]RegIIA mutant. This leads to steric hindrance between the mutant and the binding pocket. Consequently, the [P6O]RegIIA mutant completely lost its ability to inhibit the activation of rα7 nAChR in electrophysiological experiments.

Previous studies have shown that RegIIA can also act on human (h) α3β2, α3β4, and α7 nAChRs [20,25]. To further explore the potential effects of RegIIA and its variants on these human nAChRs, we performed an amino acid sequence alignment of the N-terminal extracellular domains between human and rat nAChR subunits (Figure S3). The relevant mutation sites were then mapped onto the ECD structure of the rat nAChRs. The analysis revealed that for the α3 and α7 subunits, the mutation sites are not located in the RegIIA binding region (Figure S4A,B). For the β2 subunit, only the N77T mutation is within the RegIIA binding region; however, it is not in the region where RegIIA’s H5 and P6 sites interact (Figure S4A). Therefore, we hypothesize that variants such as [H5A]RegIIA and [P6A]RegIIA should have similar effects on hα7 and hα3β2 nAChRs as they do on the corresponding rat nAChR receptors. This hypothesis is further supported by structural modeling (Figure S4D,E).

Due to significant differences between the rβ4 and hβ4 subunits at the RegIIA binding interface (Figure S4C), we conducted structural modeling of RegIIA binding to the hα3β4 nAChR and found that RegIIA binds to hα3β4 nAChR in a conformation similar to its binding with rα3β4 nAChR (Figure S4F). Further structural analysis showed that RegIIA forms 12 hydrogen bonds with hα3β4 nAChR. Within the RegIIA binding pocket, there are four amino acid differences between hα3β4 nAChR and rα3β4 nAChR (K31R, Q33E, L118K, and M160K) (Figure S4G). In the rα3β4 nAChR, K160 and E33 form three hydrogen bonds with RegIIA. However, in the interaction between hα3β4 nAChR and RegIIA, M160 and Q33 lose these hydrogen bonds, but form new hydrogen bonds at other sites (Figure S4G).

Further analysis revealed that rα3β4 nAChR and hα3β4 nAChRs have a similar H5 binding pocket for RegIIA (Figure 6). The H5 site of RegIIA does not form the hydrogen bonds with hα3β4 nAChRs that it does with rα3β4 nAChRs. This suggests that the [H5A]RegIIA variant may still maintain its interactions with hα3β4 nAChRs. Similarly, since rα3β4 nAChR and hα3β4 nAChRs have similar P6 binding pockets for RegIIA, it can be inferred that [P6A]RegIIA and other variants may lose their binding activity towards hα3β2 nAChRs.

### 2.5. Circular Dichroism Analysis of the Native RegIIA and Its Analogues

**Figure 7:**
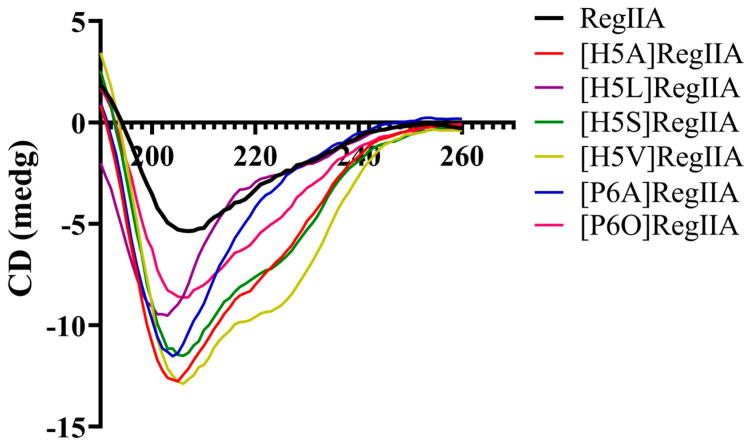
CD spectra of native RegIIA and its mutants. All CD spectra of examined peptides showed negative peaks at 208 nm and 222 nm, indicating the existence of α-helices, while the mutations H5A, H5V, and H5S made the most changes in secondary structure.

The secondary structure information of the RegIIA mutants was analyzed using circular dichroism (CD) spectra, which were then compared with that of the native peptide to determine the effect caused by the substitution of His5 and Pro6 in loop1. As shown in Figure 7, the CD spectra of all examined peptides exhibited negative peaks at 208 nm and 222 nm, suggesting the existence of α-helices. The secondary structure percentages of the mutants [H5L]RegIIA, [P6A]RegIIA, and [P6O]RegIIA were almost identical to RegIIA. Replacement of His5 with Ala, Val, and Ser led to the largest changes in secondary structure, including an approximately 10% increase in the proportion of helical structure in contrast to the native RegIIA, but an approximately 10% decrease in the proportion of sheets (Table 3).

### 2.6. Serum Stability of RegIIA and Its Analogues

**Figure 8:**
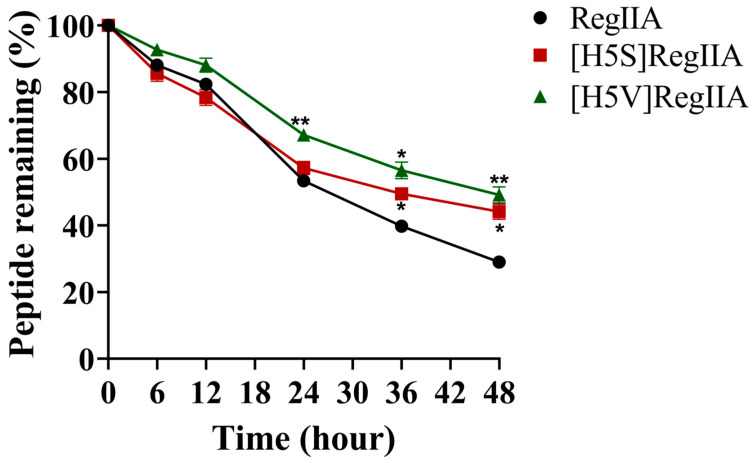
Serum stability of RegIIA and its analogues. The Stability of [H5S]RegIIA and [H5V]RegIIA vs. RegIIA were assayed in human serum. All peptides were dissolved in 100% human serum AB type and incubated at 37 °C. As the results indicated, mutations H5S and H5V led to significant improvements in stability after 48 h incubation in human serum. ** *p* < 0.01, * *p* < 0.05 (two-way ANOVA). All results are expressed as means ± SEM (*n* = 3).

The stability of RegIIA, as well as its mutants [H5S]RegIIA and [H5V]RegIIA, with improved selectivity towards rα7 nAChRs, were evaluated by incubation in human serum for 48 h at 37 °C; after which, the remaining peptides were analyzed by ultra-performance liquid chromatography (UPLC) (Figure 8). The results showed that the remaining percentage of the native RegIIA, [H5S]RegIIA, and [H5V]RegIIA were approximately 28.99%, 44.13%, and 49.17% individually after 48 h incubation in human serum, which indicated that mutations H5S and H5V significantly improved the stability of RegIIA. These results of the stability evaluation would be especially helpful for the modification of peptides for better in vivo stability, as well as for the development of specific drug delivery methods for clinical trials.

## 3. Discussion

Many native α-conotoxins lack good specificity for particular nAChR subtypes because of the high sequence homology between different nAChR subunits. To date, many efforts have been made for the structural modification of these biologically active peptides using the amino acid substitution strategy, which could lead to deeper insights into their structure–function relationship, an improvement in their potency and selectivity, as well as their further application as tool compounds and drug leads [26]. For example, alanine scanning of TxID indicated that Ser9 is important for its selectivity for rα3β4 versus rα6/α3β4 nAChRs, while [S9K]TxID only potently inhibited rα3β4 nAChRs, with no obvious effects on other subtypes [27,28]. Mutation A10L shifted the selectivity of PnIA from the rα3β2 to the rα7 subtype [29]. Optimized analogs [A7V, S9H, V10A, N11R, E14A]PeIA and [S4A, E11A, L15A]MII were more selective towards rα6/α3β2β3 over α3β2 nAChRs compared with their parent peptides, while the analog PeIA-5466 dramatically reversed the subtype preference for α3β2 over rα6/α3β2β3, which could be used to distinguish between these subtypes [30,31,32].

Previous SAR studies on α-conotoxin RegIIA have shown that the composition of loop2 notably affects its target specificity, while research on effects on activity contributed by the composition of RegIIA’s loop1 remains absent. In this study, two generations of single amino acid-substituted mutants of RegIIA were designed and synthesized, after which, their potency and selectivity towards rα3β4, rα3β2, and rα7 nAChRs were assessed. The results revealed that the amino acid residues at positions 5 and 6 in loop1 could be the determinants responsible for RegIIA’s receptor selectivity.

Many α-conotoxins, including RegIIA, have a conserved His5 in their loop1, which plays a vital role in the activity and selectivity of these peptides. Previous studies have indicated that the substitution of H5A results in completely abolished activity for LvIA and TxID, as well as a substantial potency decrease for PeIA [27,31,33]. The results in this study showed that [H5A]RegIIA had no obvious inhibition of rα3β2 and rα3β4 nAChRs, which was in agreement with previous studies on other α-conotoxins. However, its activity against rα7 nAChRs was decreased only 10-fold relative to that of RegIIA, which suggested a selectivity increase towards rα7 nAChRs. Aiming at further optimization of RegIIA and investigating the role of His5 participation in ligand–receptor interactions, we replaced the His5 of RegIIA with other amino acids including hydrophilic Gly and Ser, hydrophobic Val and Leu, and negatively charged Glu. As the results suggest, different R groups on the amino acid at position 5 may exert profound effects on RegIIA’s blockage of nAChR subtypes. All the substitutions of His5 with amino acid residues with specific chemical properties exhibited a dramatic decrease or total loss in the activity of RegIIA on rα3β2 and rα3β4 nAChRs, while the effect on the inhibition towards rα7 nAChRs was not identical. The potency of [H5E]RegIIA was comparable with that of [H5A]RegIIA, while the inhibition of [H5L]RegIIA was negligible on all three subtypes. Substitution of H5V and H5S slightly reduced the potency by two-fold towards rα7 nAChRs, resulting in a selectivity increase. It could be presumed that the side-chain properties of the 5th amino acid in loop1 may play a vital role in the molecular structure and spatial conformation of RegIIA, as well as participating in ligand–receptor interactions.

Based on our modeling results, it is evident that the H5 side chain of RegIIA forms crucial hydrogen bond interactions with α3β2 and α3β4 nAChRs. However, no hydrogen bond formation is observed in its interactions with α7 nAChRs. This finding partially explains why H5 mutants of RegIIA can better maintain the inhibition of α7 nAChR activation, while almost completely losing the ability to inhibit α3β2 and α3β4 nAChR activation. Further experimental structural evidence is essential to validate and understand the molecular mechanisms by which the H5 residue and its RegIIA mutants regulate nAChRs.

As described in previous research, Pro6 is highly conserved in the primary structure of almost all α-conotoxins, providing a backbone constraint by inducing the characteristic 3_10_ helix turn [34]. Its side chain can be fused with the main chain and form a pyrrolidine ring, which allows for the cis-trans isomerization of the Proline residue [35]. Consequently, the modification of Pro6 generally results in the disruption of the peptide structure and an inactive sequence, such as [P6A]AuIB, Vc1.1, [P6A]LvIA, etc. [33,36,37]. In contrast, introducing hydroxyproline or 5-(R)-phenyl could strengthen hydrophobic interactions with nAChRs and increase the selectivity and potency of α-conotoxins. For example, [P6O]BuIA could discriminate between α6/α3β4 and α6/α3β2β3 with a 100-fold selectivity window, while the native BuIA was only four-fold more potent on α6/α3β2β3 [19]. Substitution of Pro6 for 5-(R)-phenyl in ImI resulted in significantly increased potency against α7 nAChRs [38]. In this study, [P6A]RegIIA showed 10-fold less potency against rα7 nAChRs than native RegIIA, with no obvious inhibition against rα3β2 and rα3β4 nAChRs, leading to a >21-fold improvement in selectivity for rα7 versus both rα3β2 and rα3β4 nAChRs. Nevertheless, [P6O]RegIIA showed a complete loss in activity targeting the three receptors. The results of the CD showed that [P6A]RegIIA and [P6O]RegIIA had a similar secondary structure conformation compared with the native RegIIA, which indicated that the effect of the Pro6 substitution on RegIIA’s helical structure was not distinct. Compared to the binding pockets formed by the α3β2 and α3β4 nAChRs for the P6 residue of RegIIA, the pocket of the α7 nAChR was larger and was able to accommodate more diverse P6 mutants of RegIIA. This at least partially explains why most P6 mutants of RegIIA can still inhibit α7 nAChR activation, while almost completely losing the ability to inhibit α3β2 and α3β4 nAChR activation. Interestingly, the [P6O]RegIIA mutant, despite maintaining its secondary structure, completely loses its ability to inhibit α7 activation. Structural analysis indicates that although the α7 nAChR has a larger binding pocket for the P6 residue of RegIIA, it is still unable to accommodate [P6O]RegIIA binding. This partially explains the complete loss of inhibition of α7 activation by [P6O]RegIIA.

Given the conservation of the α3β2 and α7 nAChRs binding sites between humans and rats, along with subsequent molecular modeling analyses, it can be inferred that the corresponding variants of RegIIA are likely to exert similar effects on hα3β2 and hα7 nAChRs as they do on the rat counterparts. For α3β4 nAChRs, molecular modeling analyses suggest that [H5A]RegIIA may retain its activity on hα3β4. In contrast, [P6A]RegIIA and other variants of RegIIA at position P6 are predicted to lose their activity on hα3β4 nAChRs.

In summary, the fifth and sixth amino acid residues in loop1 were identified as the determinants responsible for the selectivity of RegIIA, while the two analogs [H5S]RegIIA and [H5V]RegIIA showed the highest increase in preference for rα7 nAChRs, with ~two-fold less potency on rα7 nAChRs and >56-fold less potency on the other two subtypes compared with the native RegIIA. These results provide deeper insights into the SAR of α-conotoxin RegIIA and lay a foundation for the optimization and modification of small peptides for better activity and stability.

## 4. Materials and Methods

### 4.1. Materials and Animals

Plasmid extraction kits and *E. coli* DH5α competent cells were purchased from Vazyme (Nanjing, China). Restriction enzymes, miniBEST DNA fragment purification kits, and DNA and RNA markers were purchased from Takara (Beijing, China). Acetonitrile (ACN), cRNA mMESSAGE mMACHINE™ transcription kits, and MEGAclear™ transcription clean-up kits were purchased from Thermo Fisher Scientific (Norristown, PA, USA). Acetylcholine (ACh), atropine, collagenase A, human serum, and bovine serum albumin (BSA) were purchased from Sigma-Aldrich (St. Louis, MO, USA). cDNAs of rat α3, α7, β2, and β4 nAChR subunits were kindly provided by S. Heinemann (Salk Institute, San Diego, CA, USA). Potassium ferricyanide (K_3_[Fe(CN)_6_]), I_2_, ascorbic acid, tris base, trifluoroacetic acid (TFA), urea, and trichloroacetic acid (TCA) were obtained from Aladdin (Shanghai, China). A preparative C18 Vydac column (10 μm, 22 mm × 250 mm) and analytical C18 Vydac column (5 μm, 4.6 mm × 250 mm) for RP-HPLC were obtained from Avantor (Radnor Township, PA, USA).

Female *Xenopus laevis* were obtained from the Kunming Institute of Zoology (Kunming, China). All animal-related experiments were conducted in accordance with ethical standards for animal research and approved by the Ethics Committee of Guangxi University (GXU-2023-0060) [39].

### 4.2. Synthesis of α-Conotoxin RegIIA and Its Analogues

The linear peptides of RegIIA and its mutants were synthesized by Motif Biotech (Suzhou, China). To obtain mature peptides with the “globular” conformation (CysI-CysIII and CysII-CysIV disulfide connectivity), Cys residues were protected in pairs with S-trityl (Trt) on CysI and CysIII and S-acetamidomethyl (Acm) on CysII and CysIV, after which, all linear peptides were oxidized by a two-step oxidation strategy, as previously described. In brief, the first disulfide bond was formed by reacting in the buffer with 20 mM K_3_[Fe(CN)_6_] and 0.1 M Tris base, pH 7.5 [33]. After this, the solution was stirred to react for 45–60 min at room temperature (~24 °C), and the monocyclic peptides with disulfide connectivity of C_I_-C_III_ were obtained. To remove the Acm protecting groups and link the second disulfide bond, the monocyclic peptides were added dropwise to 7.5 mM I_2_ dissolved in ddH_2_O/TFA/ACN (73:3:24, *v*/*v*/*v*) under nitrogen protection at room temperature (~24 °C). After a 10 min reaction, the I_2_ oxidation process was terminated by adding saturated ascorbic acid, and the mature peptides were acquired [28]. All linear, monocyclic, and bicyclic peptides were purified by preparative RP-HPLC, with a linear gradient of 5% to 50% buffer B in 30 min at a flow rate of 12 mL/min (buffer A was 0.075% TFA (*v*/*v*) in ddH_2_O, while buffer B was 0.05% TFA in 90% ACN and 10% ddH_2_O). The UV-absorbance of the peptide elution was monitored at 214 nm. The purity and quantity of the mature peptides were determined by analytical RP-HPLC, with a gradient of 5% to 50% buffer B in 30 min at a flow rate of 1 mL/min, while their molecular masses were verified by electrospray ionization-mass spectrometry (ESI-MS) [40].

### 4.3. cRNA Preparation and Injection

Plasmids containing cDNA clones of α3, α7, β2, and β4 nAChR subunits were transformed into DH5α competent cells for storage and amplification, after which, they were linearized by digestion with corresponding restriction enzymes and used as templates for cRNA preparation. The reactions for cRNA in vitro transcription were performed with mMESSAGE mMACHINE™ transcription kits for 6–10 h. Then, the products were recovered with a MEGAclear™ transcription clean-up kit.

Oocytes were acquired from female *Xenopus laevis* by operative approaches, as described previously [41]. The cRNA of the different nAChR subunits was mixed in equimolar ratios, which were then injected into oocytes, with at least 10 ng for each subunit per oocyte within 24 h following oocyte harvest. The injected oocytes were then incubated at 17 °C at a humidity of 35% in ND96 solution (96 mM NaCl, 2 mM KCl, 1.8 mM CaCl_2_, 1 mM MgCl_2_, 5 mM HEPES, pH 7.1–7.5), with antibiotics (10 mg/L penicillin, 10 mg/L streptomycin, 100 mg /L gentamicin).

### 4.4. Electrophysiology

All electrophysiological experiments were operated at room temperature (~24 °C). The ACh-evoked currents mediated by nAChRs expressed on the membrane of oocytes were recorded using a two-electrode voltage clamp amplifier (OC-725D, WARNER Instrument, Holliston, MA, USA) and a digital-to-analog converter (Dendrite, Sutter Instrument, Novato, CA, USA) 2–5 days after cRNA injection, as previously described [33,40,42]. The electrodes for voltage clamping and current recording were pulled from borosilicate glass, whose resistance was about 0.5–2 MΩ when filled with 3 M KCl. The oocytes were placed in a cylindrical chamber with a volume of approximately 50 μL and clamped at a holding potential of −70 mV. Gravity perfusion was then conducted with ND96 solution containing 0.1 mg/mL BSA and 1 μM atropine (exception for rα7 nAChR) at a flow rate of 2–4 mL/min. During the electrophysiological data recording period, the oocytes were supplied with ACh pulses for 2 s once per minute, followed by 58 s washout with ND96 solution. The ACh concentration was 200 μM for rα7 nAChR, while 100 μM for the rα3β2 and rα3β4 subtypes (the EC_50_ values of agonist ACh on rα3β2, rα3β4, and rα7 nAChRs were 104 μM, 110 μM, and 290 μM, respectively) [43]. The oocytes expressing nAChR subtypes were incubated with ND96 solution for 5 min and the current values induced by ACh pulse were then obtained as the control response. The blocking effects of RegIIA and its mutants were determined by comparing the ACh-induced current amplitudes after 5 min incubation with different concentrations of peptide analogs to the control current.

### 4.5. Date Analysis

All concentration–response curves and half inhibiting concentrations (IC_50_s) of the antagonists were obtained by fitting the electrophysiological data to a nonlinear-regression equation using Graphpad Prism 6.0 (GraphPad Software, San Diego, CA, USA): response% = 100/[1 + ([toxin]/IC_50_)^nH^], in which nH represents the Hill coefficient. Each point of the concentration–response curves represents the average value ± standard error of the mean (SEM) of replicated data from 6–8 oocytes.

### 4.6. Circular Dichroism Spectroscopy

CD spectra of RegIIA and its analogues were obtained using a J-810 CD spectropolarimeter (Jasco Corp., Tokyo, Japan) at room temperature (~24 °C). All peptides were dissolved in ddH_2_O, respectively. The spectral data of peptides were recorded over a wavelength range of 190–260 nm, with a data pitch and band width of 1 nm. The scanning speed was 200 nm/min. The spectra were obtained from three replicates and the secondary structure of the peptides was calculated using the CDSSTR method.

### 4.7. Serum Stability Assay

Human Male AB serum was pretreated as according to a previous study [44]. The peptides were dissolved in serum at a concentration of 100 μM and incubated at 37 °C. The aliquots were taken out at time points 0, 6, 12, 24, 36, 48 h post-incubation, after which, an equal volume of 6 M urea was added to the aliquots and incubated at 4 °C for 10 min to denature the protein. Following denaturation, an equal volume of 20% TCA was added and incubated for precipitation. Then, the samples were centrifuged at 14,000 rpm for 15 min, and the supernatant was collected and analyzed by UPLC. The stability was calculated as the peak area of the remaining peptide at each time point as a percentage of the peak area at 0 h. The stability assay experiment for each peptide was performed at least 3 times.

### 4.8. AlphaFold 3-Based Molecular Modeling

Molecular modeling was carried out using the AlphaFold 3 server [21]. The RegIIA amino acid sequence and the extracellular domain (ECD) amino acid sequences of the rat α3, β2, β4, and α7 subunits were used for modeling the structures of the complexes of RegIIA and its mutants bound to α3β2, α3β4, and α7, respectively. The same molecular modeling approach was used for the corresponding human nAChRs. The quality of the model was validated with the SAVES v6.1 server (https://saves.mbi.ucla.edu/ (accessed on 10 July 2024)), and all of the structural figures were made with PyMOL (http://www.pymol.org/ (accessed on 23 October 2022)).

## Figures and Tables

**Table 1:** Potencies of RegIIA and its loop1 alanine scanning mutants on rα3β2, rα3β4, and rα7 nAChR subtypes.

Peptides	rα3β2		rα3β4		rα7	
	IC_50_ (nM) ^a^	Hillslope ^a^	IC_50_ (nM) ^a^	Hillslope ^a^	IC_50_ (nM) ^a^	Hillslope ^a^
RegIIA	36 (28–44)	1.0 (0.8–1.2)	102 (78–129)	0.9 (0.8–1.2)	43 (26–62)	0.8 (0.6–1.0)
[G1A]RegIIA	90 (51–138)	0.7 (0.5–0.9)	132 (102–165)	1.0 (0.8–1.3)	286 (226–364)	1.1 (0.9–1.4)
[S4A]RegIIA	34 (26–42)	0.9 (0.7–1.0)	95 (73–121)	0.9 (0.7–1.1)	39 (25–56)	0.8 (0.6–1.0)
[H5A]RegIIA	>10,000 ^b^	ND ^c^	>10,000 ^b^	ND ^c^	446 (327–574)	1.0 (0.8–1.4)
[P6A]RegIIA	>10,000 ^b^	ND ^c^	>10,000 ^b^	ND ^c^	459 (317–617)	1.2 (0.8–2.2)
[A7G]RegIIA	>10,000 ^b^	ND ^c^	964 (814–1152)	1.1 (0.9–1.5)	600 (488–731)	0.9 (0.8–1.1)

^a^ IC_50_ values with 95% confidence interval; ^b^ Less than 50% block at 10 μM; ^c^ ND, not determined.

**Table 2:** Potencies of RegIIA’s second-generation mutants on rα3β2, rα3β4, and rα7 nAChR subtypes.

Peptides	rα3β2		rα3β4		rα7	
	IC_50_ (nM) ^a^	Hillslope ^a^	IC_50_ (nM) ^a^	Hillslope ^a^	IC_50_ (nM) ^a^	Hillslope ^a^
[H5S]RegIIA	>10,000 ^b^	ND ^c^	5732 (3975–8006)	0.8 (0.6–1.0)	100 (76–128)	0.9 (0.7–1.1)
[H5V]RegIIA	>10,000 ^b^	ND ^c^	8800 (6094–12,240)	0.9 (0.7–1.4)	97 (77–119)	1.0 (0.8–1.3)
[H5L]RegIIA	>10,000 ^b^	ND ^c^	>10,000 ^b^	ND ^c^	>10,000 ^b^	ND ^c^
[H5G]RegIIA	>10,000 ^b^	ND ^c^	>10,000 ^b^	ND ^c^	3539 (2846–4416)	1.2 (1.0–1.4)
[H5E]RegIIA	>10,000 ^b^	ND ^c^	>10,000 ^b^	ND ^c^	441 (335–552)	1.1 (0.9–1.5)
[P6O]RegIIA	>10,000 ^b^	ND ^c^	>10,000 ^b^	ND ^c^	>10,000 ^b^	ND ^c^

^a^ IC_50_ values with 95% confidence interval; ^b^ Less than 50% block at 10 μM; ^c^ ND, not determined.

**Table 3:** Secondary structure percentages of the native RegIIA and its mutants.

Peptides	α-Helix (%)	β-Sheet (%)	β-Turn (%)	Random Coil (%)
RegIIA	10%	31%	25%	33%
[H5A]RegIIA	19%	21%	26%	35%
[H5L]RegIIA	9%	31%	25%	34%
[H5S]RegIIA	18%	22%	26%	34%
[H5V]RegIIA	20%	20%	28%	32%
[P6A]RegIIA	12%	30%	24%	33%
[P6O]RegIIA	13%	29%	25%	34%

## Data Availability

The data presented in this study are available in the article.

## Supplementary Materials

The following supporting information can be downloaded at: https://www.mdpi.com/article/10.3390/md22090390/s1, Figure S1: RP-HPLC analysis of RegIIA and its mutants; Figure S2: ESI-MS analysis of RegIIA and its mutant; Figure S3: Amino acid sequence alignment of the N-terminal extracellular domains of rat and human α7, as well as α3, β2, and β4 nAChR subunits; Figure S4: Interactions between RegIIA and its mutants with the ECDs of hα3β2, hα3β4, and hα7 nAChRs; additional figures illustrating the RP-HPLC and ESI-MS analysis of RegIIA and its mutants (Figures S1 and S2).
