# Supplementary material for: Single Amino Acid Substitution in Loop1 Switches the Selectivity of α-Conotoxin RegIIA towards the α7 Nicotinic Acetylcholine Receptor
Source: Mar Drugs. 2024 Aug 29;22(9):390. doi: 10.3390/md22090390 (PMC11433573; doi:10.3390/md22090390)
Supplement: Supplementary file 1 [file marinedrugs-22-00390-s001.zip › Single Amino Acid Substitution in Loop1 Switches the Selectivity of a┴-Conotoxin RegIIA towards a┴7 Nicotinic Acetylcholine Receptor+supplementary+files.pdf]

Supporting Information

Single Amino Acid Substitution in Loop 1 Switches the Selectivity of α-Conotoxin RegIIA towards α7 Nicotinic Acetylcholine Receptor

Jinpeng Yu[1,⊥,*], Junjie Xie[⊥,1], Yuting Ma[1], Pengcheng Wei[1], Panpan Zhang[1], Zepei Tang[1], Xiaopeng Zhu[1], Dongting Zhangsun[1,2] and Sulan Luo[1,2,*]

[1] Guangxi Key Laboratory of Special Biomedicine; School of Medicine, Guangxi University, Nanning, 530004, China
[2] Key Laboratory of Tropical Biological Resources of Ministry of Education, Hainan University, Haikou 570228, China
[*] Correspondence: yujinpeng@gxu.edu.cn (J.Y.); Sulan2021@gxu.edu.cn (S.L.)
[⊥] Co-first author: yujinpeng@gxu.edu.cn (J.Y.); 2128391011@st.gxu.edu.cn (J.X.)

Table of Contents

Figure S1. RP-HPLC analysis of RegIIA and its mutants. (Page S2)

Figure S2. ESI-MS analysis of RegIIA and its mutants. (Page S3)

Figure S3. Amino acid sequence alignment of the N-terminal extracellular domains of rat and human α7, as well as α3, β2 and β4 nAChR subunits. (Page S4)

Figure S4. Interactions between RegIIA and its mutants with the ECDs of hα3β2, hα3β4, and hα7 nAChRs. (Page S5)

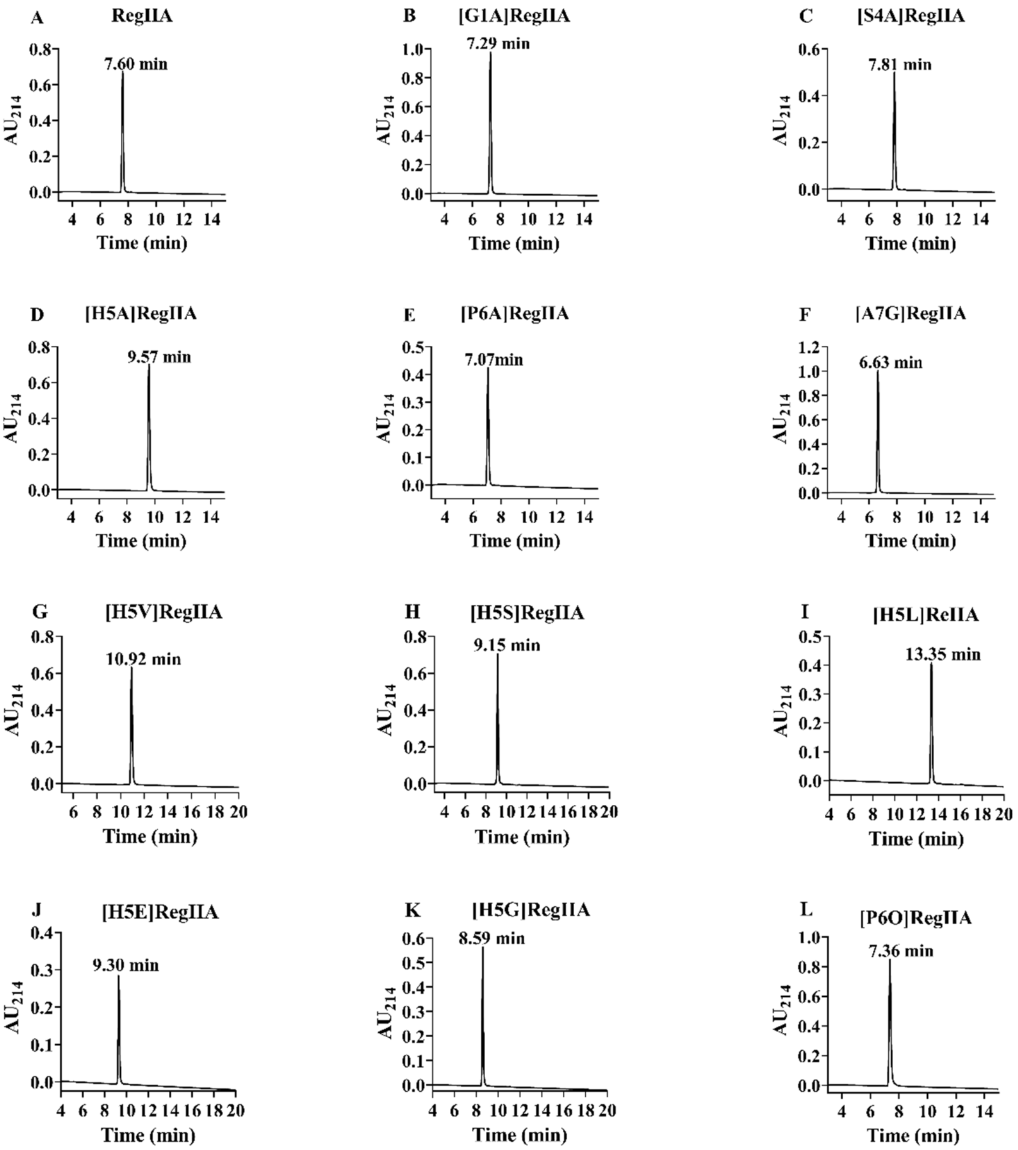

Figure S1. RP-HPLC analysis of RegIIA and its mutants (A-L).

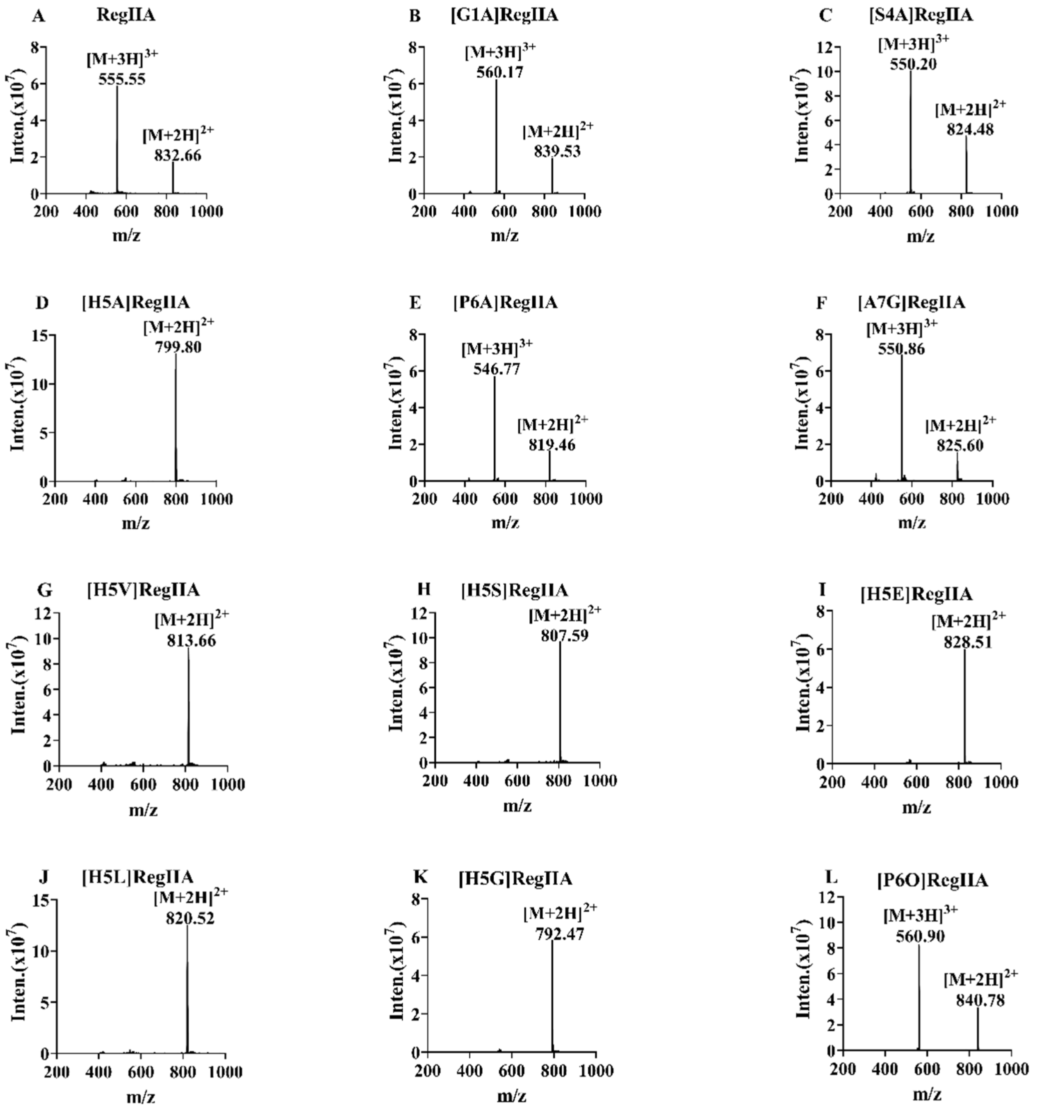

Figure S2. ESI-MS analysis of RegIIA and its mutants (A-L).

A

```
                                20                    40                    60
rα7 GEFQRRLYKE LVKNYNPLER PVANDSQPLT VYFSLSLLQI MDVDEKNQVL TTNIWLQMSW 60
hα7 GEFQRKLYKE LVKNYNPLER PVANDSQPLT VYFSLSLLQI MDVDEKNQVL TTNIWLQMSW 60
                                80                    100                   120
rα7 TDHYLQWNMS EYPGVKNVRF PDGQIWKPDI LLYNSADERF DATFHTNVLV NASGHCQYLP 120
hα7 TDHYLQWNVS EYPGVKTVRF PDGQIWKPDI LLYNSADERF DATFHTNVLV NSSGHCQYLP 120
                                140                   160                   180
rα7 PGIFKSSCYI DVRWFPFDVQ QCKLKFGSWS YGGWSLDLQM QEADISSYIP NGEWDLMGIP 180
hα7 PGIFKSSCYI DVRWFPFDVQ HCKLKFGSWS YGGWSLDLQM QEADISGYIP NGEWDLVGIP 180
                                200
rα7 GKRNEKFYEC CKEPYPDVTY TVTMRRRT 208
hα7 GKRSERFYEC CKEPYPDVTF TVTMRRRT 208
```

B

```
                                20                    40                    60
rα3 EHRLFQYLFE DYNEIIRPVA NVSHPVIIQF EVSMSQLVKV DEVNQIMETN LWLKQIWNDY 60
hα3 EHRLFERLFE DYNEIIRPVA NVSDPVIIHF EVSMSQLVKV DEVNQIMETN LWLKQIWNDY 60
                                80                    100                   120
rα3 KLKWKPSDYQ GVEFMRVPAE KIWKPDIVLY NNADGDFQVD DKTKALLKYT GEVTWIPPAI 120
hα3 KLKWNPSDYG GAEFMRVPAQ KIWKPDIVLY NNAVGDFQVD DKTKALLKYT GEVTWIPPAI 120
                                140                   160                   180
rα3 FKSSCKIDVT YFPFDYQNCT MKFGSWSYDK AKIDLVLIGS SMNLKDYWES GEWAIIKAPG 180
hα3 FKSSCKIDVT YFPFDYQNCT MKFGSWSYDK AKIDLVLIGS SMNLKDYWES GEWAIIKAPG 180
                                200
rα3 YKHEIKYNCC EEIYQDITYS LYIRRLP 207
hα3 YKHDIKYNCC EEIYPDITYS LYIRRLP 207
```

C

```
                                20                    40                    60
rβ2 EERLVEHLLD PSRYNKLIRP ATNGSELVTV QLMVSLAQLI SVHEREQIMT TNVWLTQEWE 60
hβ2 EERLVEHLLD PSRYNKLIRP ATNGSELVTV QLMVSLAQLI SVHEREQIMT TNVWLTQEWE 60
                                80                    100                   120
rβ2 DYRLTWKPED FDNMKKVRLP SKHIWLPDVV LYNNADGMYE VSFYSNAVVS YDGSIFWLPP 120
hβ2 DYRLTWKPEE FDNMKKVRLP SKHIWLPDVV LYNNADGMYE VSFYSNAVVS YDGSIFWLPP 120
                                140                   160                   180
rβ2 AIYKSACKIE VKHFPFDQQN CTMKFRSWTY DRTEIDLVLK SDVASLDDFT PSGEWDIIAL 180
hβ2 AIYKSACKIE VKHFPFDQQN CTMKFRSWTY DRTEIDLVLK SEVASLDDFT PSGEWDIVAL 180
                                200
rβ2 PGRRNENPDD STYVDITYDF IIRRKP 206
hβ2 PGRRNENPDD STYVDITYDF IIRRKP 206
```

D

```
                                20                    40                    60
rβ4 EEKLMDDLLN KTRYNNLIRP ATSSSQLISI RLELSLSQLI SVNEREQIMT TSIWLKQEWT 60
hβ4 EEKLMDDLLN KTRYNNLIRP ATSSSQLISI KLQLSLAQLI SVNEREQIMT TNVWLKQEWT 60
                                80                    100                   120
rβ4 DYRLAWNSSC YEGVNILRIP AKRVWLPDIV LYNNADGTYE VSVYTNVIVR SNGSIQWLPP 120
hβ4 DYRLTWNSSR YEGVNILRIP AKRIWLPDIV LYNNADGTYE VSVYTNLIVR SNGSVLWLPP 120
                                140                   160                   180
rβ4 AIYKSACKIE VKHFPFDQQN CTLKFRSWTY DHTEIDMVLK SPTAIMDDFT PSGEWDIVAL 180
hβ4 AIYKSACKIE VKYFPFDQQN CTLKFRSWTY DHTEIDMVLM TPTASMDDFT PSGEWDIVAL 180
                                200
rβ4 PGRRTVNPQD PSYVDVTYDF IIKRKP 206
hβ4 PGRRTVNPQD PSYVDVTYDF IIKRKP 206
```

Figure S3. Amino acid sequence alignment of the N-terminal extracellular domains of rat and human α7 (A), as well as α3 (B), β2 (C) and β4 (D) nAChR subunits.

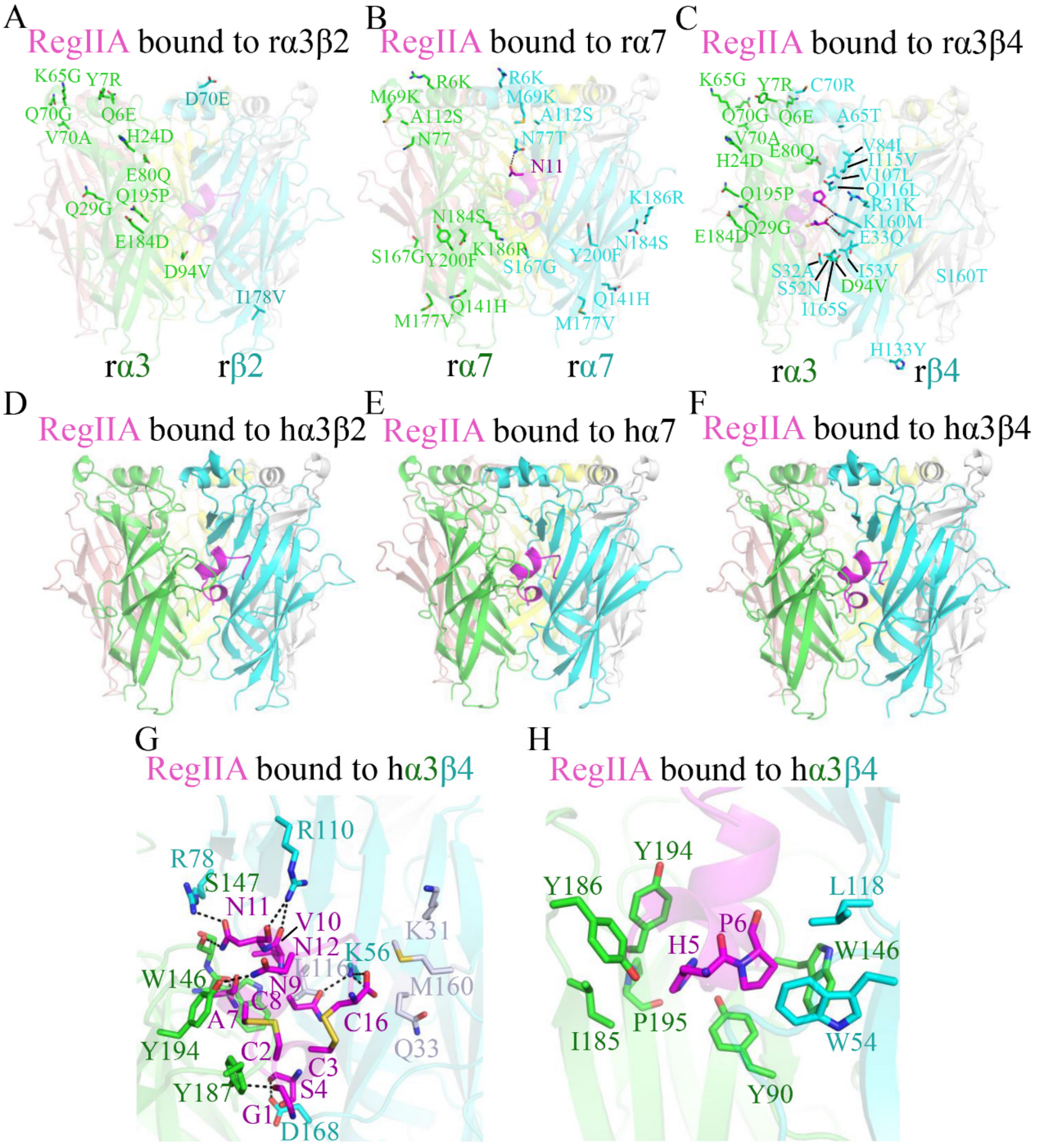

Figure S4. Interactions between RegIIA and its mutants with the ECDs of hα3β2, hα3β4, and hα7 nAChRs. (A) The different amino acids between human and rat α3β2 nAChRs are displayed in the ECD structure of rα3β2. (B) The differences between human and rat α3β4 nAChRs are shown in the ECD structure of rα3β4. (C) The differences between human and rat α7 nAChRs are shown in the ECD structure of rα7. (D) Overall structure of RegIIA binding to the ECD of hα3β2 nAChR. (E) Overall structure of RegIIA binding to the ECD of hα7 nAChR. (F) Overall structure of RegIIA binding to the ECD of hα3β4 nAChR. (G) Detailed interactions of RegIIA with the hα3β4 nAChR, showing all hydrogen bonds. Disulfide bonds between the side chains of C2-C8 and C3-C16 are shown. (H) Detailed interactions of RegIIA's H5 and P6 with hα3β2 nAChR.
