# Supplementary figures and images for: Single Amino Acid Substitution in Loop1 Switches the Selectivity of α-Conotoxin RegIIA towards the α7 Nicotinic Acetylcholine Receptor
Source: Mar Drugs. 2024 Aug 29;22(9):390. doi: 10.3390/md22090390 (PMC11433573; doi:10.3390/md22090390)

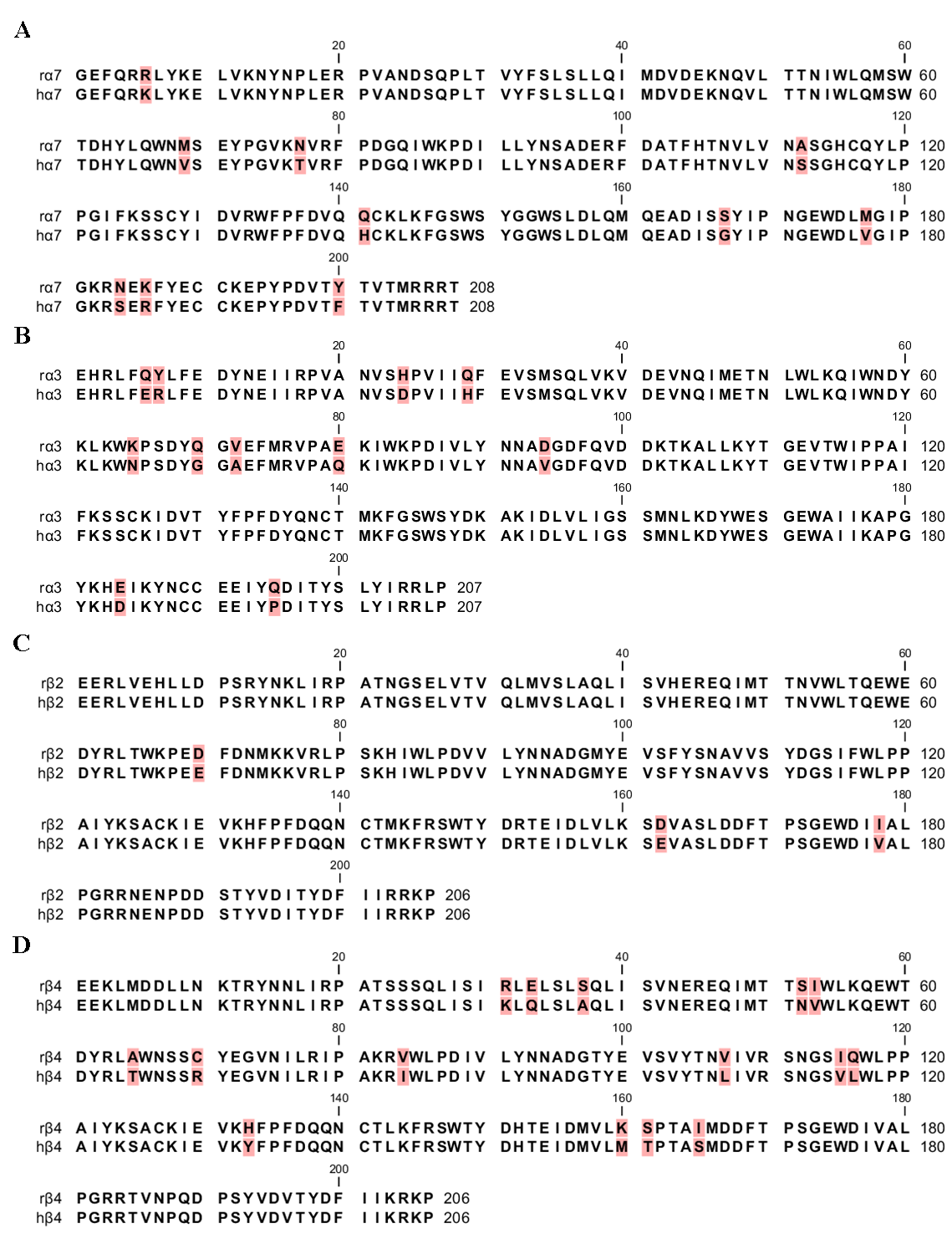

Supplement: Supplementary file 1 [file marinedrugs-22-00390-s001.zip › Figure S3.tif]

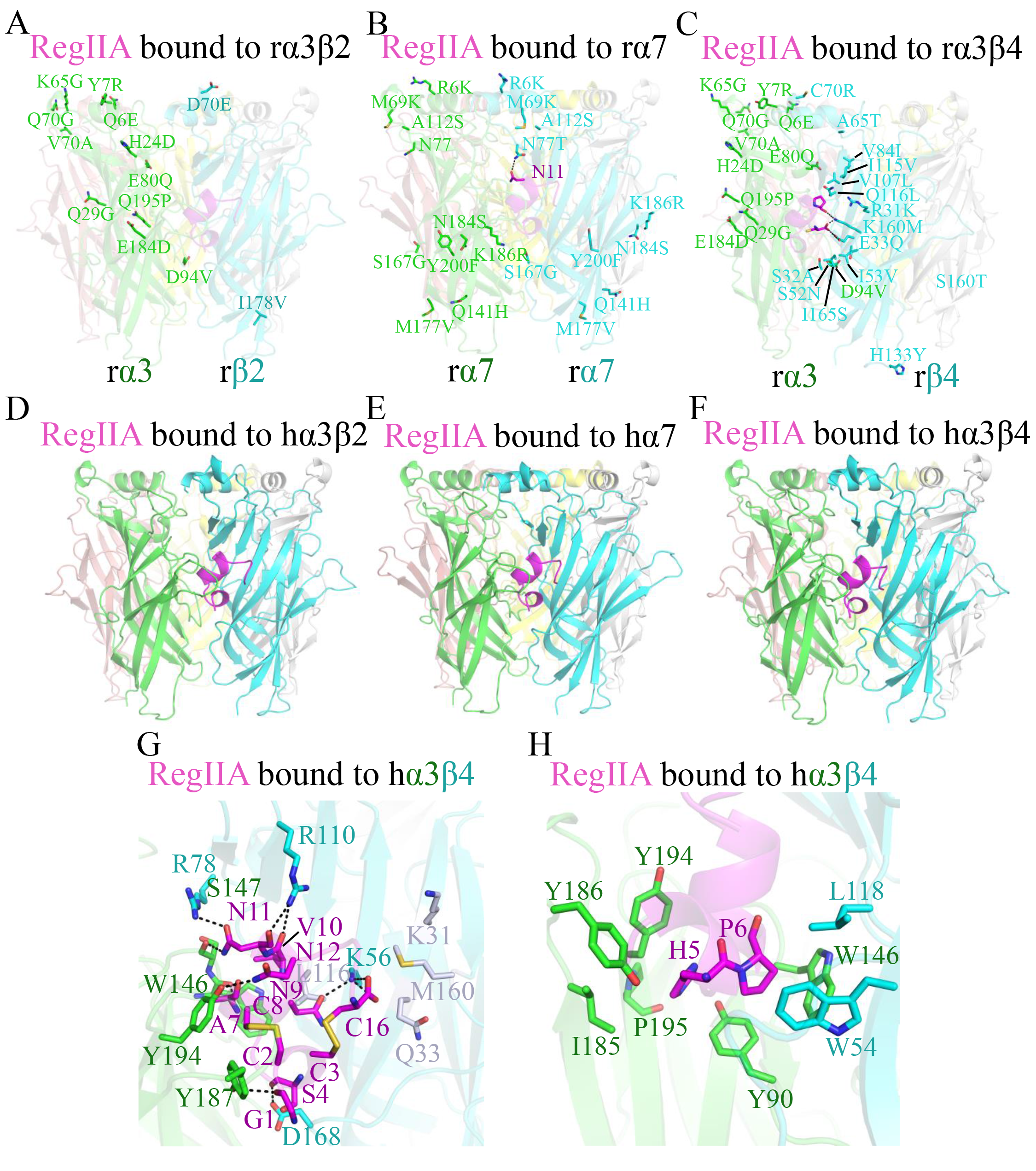

Supplement: Supplementary file 1 [file marinedrugs-22-00390-s001.zip › Figure S4.tif]
